# c-Src-dependent EGF receptor transactivation contributes to ET-1-induced COX-2 expression in brain microvascular endothelial cells

**DOI:** 10.1186/1742-2094-9-152

**Published:** 2012-07-02

**Authors:** Hsi-Lung Hsieh, Chih-Chung Lin, Hui-Ju Chan, Caleb M Yang, Chuen-Mao Yang

**Affiliations:** 1 Department of Nursing, Division of Basic Medical Sciences, Chang Gung University of Science and Technology, Tao-Yuan, Taiwan; 2 Department of Anesthetics, Chang Gung Memorial Hospital and College of Medicine, Chang Gung University, Kwei-San, Tao-Yuan, Taiwan; 3 Department of Physiology and Pharmacology, and Health Aging Research Center, College of Medicine, Chang Gung University, Tao-Yuan, Taiwan; 4 School of Medicine, National Yang Ming University, Taipei, Taiwan; 5 Department of Pharmacology, Chang Gung University, 259 Wen-Hwa 1st Road, Kwei-San, Tao-Yuan, Taiwan

## Abstract

**Background:**

Endothelin-1 (ET-1) is elevated and participates in the regulation of several brain inflammatory disorders. The deleterious effects of ET-1 on endothelial cells may aggravate brain inflammation mediated through the upregulation of cyclooxygenase-2 (COX-2) gene expression. However, the signaling mechanisms underlying ET-1-induced COX-2 expression in brain microvascular endothelial cells remain unclear.

**Objective:**

The goal of this study was to examine whether ET-1-induced COX-2 expression and prostaglandin E_2_ (PGE_2_) release were mediated through a c-Src-dependent transactivation of epidermal growth factor receptor (EGFR) pathway in brain microvascular endothelial cells (bEnd.3 cells).

**Methods:**

The expression of COX-2 induced by ET-1 was evaluated by Western blotting and RT-PCR analysis. The COX-2 regulatory signaling pathways were investigated by pretreatment with pharmacological inhibitors, short hairpin RNA (shRNA) or small interfering RNA (siRNA) transfection, chromatin immunoprecipitation (ChIP), and promoter activity reporter assays. Finally, we determined the PGE_2_ level as a marker of functional activity of COX-2 expression.

**Results:**

First, the data showed that ET-1-induced COX-2 expression was mediated through a c-Src-dependent transactivation of EGFR/PI3K/Akt cascade. Next, we demonstrated that ET-1 stimulated activation (phosphorylation) of c-Src/EGFR/Akt/MAPKs (ERK1/2, p38 MAPK, and JNK1/2) and then activated the c-Jun/activator protein 1 (AP-1) via G_q/i_ protein-coupled ET_B_ receptors. The activated c-Jun/AP-1 bound to its corresponding binding sites within COX-2 promoter, thereby turning on COX-2 gene transcription. Ultimately, upregulation of COX-2 by ET-1 promoted PGE_2_ biosynthesis and release in bEnd.3 cells.

**Conclusions:**

These results demonstrate that in bEnd.3 cells, c-Src-dependent transactivation of EGFR/PI3K/Akt and MAPKs linking to c-Jun/AP-1 cascade is essential for ET-1-induced COX-2 upregulation. Understanding the mechanisms of COX-2 expression and PGE_2_ release regulated by ET-1/ET_B_ system on brain microvascular endothelial cells may provide rational therapeutic interventions for brain injury and inflammatory diseases.

## Background

Cyclooxygenase (COX) is a rate-limiting key enzyme in the synthesis of prostaglandins (PGs) and thromboxane. In this process, phospholipase A_2_ catalyzes the release of arachidonic acid (AA) from membrane phospholipids, while COX catalyzes the conversion of AA into PGH_2_, which is the common precursor of all prostanoids [1,2]. Two COX isoforms have been demonstrated: COX-1, which is constitutively expressed in most tissues, regulates normal physiological responses and controls renal and vascular homeostasis; COX-2, another COX isoform, is not detectable in most normal tissues or resting cells, but its expression can be induced by various stimuli, including cytokines, endotoxin, and growth factors to produce proinflammatory PGs during inflammatory responses in several cell types including vascular endothelial and smooth muscle cells [3,4]. Previous studies have shown that COX-2 immunoreactivity is detected in various inflammatory tissues, including synovial macrophage and vascular cells of patients with arthritis and atherosclerosis, respectively. Several lines of evidence have further confirmed COX-2 as a major therapeutic target for the treatment of inflammatory disorders such as arthritis [1]. Moreover, homozygous deletion of the COX-2 gene in mice leads to a striking reduction of endotoxin-induced inflammation [5]. Therefore, COX-2 may play an important role in the development of various inflammatory responses such as vascular inflammation (i.e., atherosclerosis and hypertension). In brain, upregulation of COX-2 leads to increased production of PGs, which are potent inflammatory mediators associated with neurodegenerative disorders [6]. Thus, COX-2 and its metabolites PGs may act as a major pathological factor in brain inflammatory diseases.

The endothelium plays an important role in the regulation of vascular function by producing a large number of biologically active substances that participate in the regulation of vascular functions. In brain, cerebral capillary and microvascular endothelial cells play an active role in maintaining cerebral blood flow, microvascular tone, and blood–brain barrier (BBB) functions [7]. Dysfunction of the vascular endothelium is an early finding in the development of various vascular diseases and is closely related to clinical events in patients with atherosclerosis and hypertension [8,9]. Endothelial cells are known to produce vasoactive mediators such as endothelin (ET) to maintain hemodynamic responses. Among the ET family, the bioactivity of ET-1 is mediated through potent vasoconstrictor and proinflammatory action, and has been implicated in the pathogenesis of hypertension and vascular diseases [9-11]. Two types of ET receptors, ET type A (ET_A_) and type B (ET_B_), are responsible for ET-1-triggered biological effects, which are mediated via G protein*-*dependent regulation [12]. In the central nervous system (CNS), ET-1 also plays a substantial role in the normal development or in CNS diseases. Both endothelial cells [13] and astrocytes [14] are potential sources of ET-1 release in response to hypoxic/ischemic injury of the brain. The ET_B_ receptors are located on both endothelial and vascular smooth muscle cells, and modulate post-injury responses of these cells in the CNS [11]. There has been an increasing interest in the regulatory role of endothelial cells in neurovascular coupling, which matches an adequate supply of cerebral blood flow with the local metabolic demands that are imposed by neural activity [15]. As a fundamental component of the neurovascular unit, endothelium dysfunction has been implicated in neurodegenerative diseases [15,16]. Circumstantial evidence has further demonstrated that overexpression of ET-1 on endothelial cells has deleterious effects on ischemic brain [7]. Endothelial ET-1 can induce cytokine or chemokine (e.g., interleukin-1 or interleukin-8) production and secretion by non-neuronal cells, including astrocytes and endothelial cells, which directly contribute to BBB breakdown during CNS inflammation [17]. These findings imply the involvement of ET-1 in neuroinflammation in the CNS. However, the detailed mechanisms responsible for ET-1 action remain unclear.

ET-1 activates multiple signaling pathways and regulates diverse cellular functions via ET receptors (ET_A_ or ET_B_), which couple to various G proteins such as G_q_ and G_i_[12,18-20]. The principal mechanism underlying activation by ET-1 is mediated through ET_B_ receptors coupling G_q_ proteins, resulting in activation of phospholipase C (PLC)-β, phosphoinositide (PI) hydrolysis, and formation of inositol trisphosphate (IP_3_) and diacylglycerol, leading to Ca^2+^ increase and protein kinase C (PKC) activation [21]. Activation of ET_B_ receptor has been also shown to inhibit adenylyl cyclase via coupling to G_i_ proteins [22]. Several lines of evidence demonstrate that mitogen-activated protein kinases (MAPKs) could be activated by the activation of G_q_ and G_i_ protein-coupled receptors via different signal pathways [23]. MAPKs activation by ET-1 has been shown to modulate various cellular responses, including cellular hypertrophy, growth, proliferation, and cell survival in various cell types [19,24]. Induction of COX-2 expression requires activation of MAPK and stimulation of particular transcription factors in various cell types [20,25,26]. Moreover, it has been shown that signaling through MAPKs, extracellular signal-regulated protein kinase 1/2 (ERK1/2) especially, in response to GPCR agonists can be mediated through transactivation of the epidermal growth factor receptor (EGFR) [27]. The transactivation of EGFR by GPCRs mediated by activation of non-receptor tyrosine kinases such as the Src family or release of heparin-binding EGF-like growth factor (HB-EGF) has been demonstrated in various cell types [28]. ET-1 has also been shown to share this transactivation of EGFR in ovarian cancer cells or VSMCs, leading to MAPK activation and then regulating cell proliferation or COX-2 expression, respectively [29,30]. Our previous report demonstrated that bradykinin stimulates ERK1/2 activation and cell proliferation via Src family kinases and EGFR transactivation in VSMCs [31]. Additionally, ET-1 can stimulate transactivation of EGFR via ET_A_ receptors in rat cardiac fibroblasts [32]. Several previous reports have also demonstrated that GPCR agonists (e.g., sphingosine 1-phosphate and thrombin) stimulate ERK1/2 phosphorylation and AP-1 activation associated with COX-2 expression in rat VSMCs [4,31]. However, several reports have demonstrated that proinflammatory stimuli, which play a critical role in inflammation, rapidly upregulate AP-1-dependent genes such as COX-2 [33-35]. In brain microvascular endothelial cells, the mechanisms underlying ET-1-induced COX-2 expression and PGE_2_ production are not completely defined, the c-Src-dependent transactivation of EGFR cascade especially.

In this study, we investigated the molecular mechanisms underlying ET-1-induced COX-2 expression in mouse brain microvascular endothelial (bEnd.3) cells. These findings suggested that ET-1 induces COX-2 expression at the transcriptional and translational levels, which is mediated through the ET_B_ receptor (coupling to G_i_ and G_q_)-mediated c-Src-dependent transactivation of EGFR and activation of PI3K/Akt, ERK1/2, p38 MAPK, JNK1/2, and c-Jun/AP-1 pathways, leading to PGE_2_ biosynthesis in mouse bEnd.3 cells. These results provide new insights into the mechanisms of ET-1 action, which may be therapeutic targets in brain inflammatory diseases.

## Methods

### Materials

Dulbecco’s modified Eagle’s medium (DMEM)/F-12 medium, fetal bovine serum (FBS), and TRIzol were from Invitrogen (Carlsbad, CA). The Hybond C membrane and enhanced chemiluminescence (ECL) Western blot detection system were from GE Healthcare Biosciences (Buckinghamshire, UK). Anti-COX-2 monoclonal antibody was from BD Transduction Laboratories (San Diego, CA). Phospho-c-Src (#2120), Phospho-EGFR (#2231), Phospho-Akt (#9271), Phospho-ERK1/2 (#9101), Phospho-p38 (#9211), Phospho-JNK1/2 (#9255), and Phospho-c-Jun (#2361) antibodies were from Cell Signaling (Danver, MA). c-Src (sc-8056), EGFR (sc-03), p85 (sc-423), Akt (sc-8312), and c-Jun (sc-1694) antibodies were from Santa Cruz (Santa Cruz, CA). Anti-glyceraldehyde-3-phosphate dehydrogenase (GAPDH, cat. no. 4699–9555) antibody was from Biogenesis (Boumemouth, UK). Genistein, PP1, AG1478, LY294002, SH-5, BQ-123, BQ-788, GP antagonist-2, GP antagonist-2A, U0126, SB202190, SP600125, and tanshinone IIA were from Biomol (Plymouth Meeting, PA). Bicinchoninic acid (BCA) protein assay reagent was from Pierce (Rockford, IL). Enzymes, ET-1, and other chemicals were from Sigma (St. Louis, MO).

### Mouse brain microvascular endothelial cell culture

Mouse brain microvascular endothelial cells (bEnd.3) were purchased from Bioresource Collection and Research Centre (BCRC, Hsinchu, Taiwan) and were grown in DMEM/F-12 containing 10% FBS and antibiotics (100 U/ml penicillin G, 100 μg/ml streptomycin, and 250 ng/ml fungizone) at 37 °C in a humidified 5% CO_2_ atmosphere. When the cultures had grown to confluence, cells were released with 0.05% (w/v) trypsin/0.53 mM EDTA for 5 min at 37 °C. The cell suspension was diluted with DMEM/F-12 containing 10% FBS to a concentration of 2 × 10^5^ cells/ml. The cell suspension was plated onto 6-well culture plates (2 ml/well) or 10-cm culture dishes (10 ml/dish) for the measurement of protein or RNA expression, respectively. Culture medium was changed after 24 h and then every 3 days. Experiments were performed with cells from passages 5 to 13.

### Preparation of cell extracts and Western blot analysis

Growth-arrested cells were incubated with ET-1 at 37 °C for various time intervals. The cells were washed with ice-cold phosphate-buffered saline (PBS), scraped, and collected by centrifugation at 45,000 × g for 1 h at 4 °C to yield the whole cell extract, as described previously [4]. Samples were denatured, subjected to SDS-PAGE using a 10% (w/v) running gel, and transferred to nitrocellulose membrane. Membranes were incubated overnight using an anti-COX-2, Phospho-c-Src, Phospho-EGFR, Phospho-Akt, Phospho-ERK1/2, Phospho-p38, Phospho-JNK1/2, and Phospho-c-Jun, c-Src, EGFR, p85, Akt, c-Jun, or GAPDH antibody (1:1,000 dilution). Membranes were washed with TTBS four times for 5 min each, incubated with a 1:2,000 dilution of anti-rabbit horseradish peroxidase antibody for 1 h. The immunoreactive bands were detected by ECL reagents.

### Total RNA extraction and gene expression

For reverse transcription PCR (RT-PCR) analysis, total RNA was extracted from mouse brain endothelial cells stimulated by ET-1, as previously described [4]. The cDNA obtained from 0.5 μg total RNA was used as a template for PCR amplification. Oligonucleotide primers were designed based on Genbank entries for mouse COX-2 and β-actin. The following primers were used for amplification reaction: for COX-2: 5′-(AAAACCGTGGGGAATGTATGAGC)-3′ (sense), 5′-(GATGGGTGAAGTGCTGGGGAAAG)-3′ (anti-sense); for β-actin: 5′-(GAACCCTAAGGCCAACCGTG)-3′ (sense), 5′-(TGGCATAGAGGTCTTTACGG)-3′ (anti-sense). PCR mixes contained 10 μl of 5× PCR buffer, 1.25 mM of each dNTP, 100 pmol of each forward and reverse primer, and 2.5 units of Taq polymerase (Takara, Shiga, Japan). The final reaction volume was 50 μl. Amplification was performed in 25 cycles at 94 °C, 20 s; 60 °C, 40 s; 72 °C, 40 s [36]. After the last cycle, all samples were incubated for an additional 10 min at 72 °C. PCR fragments were analyzed on 2% agarose 1× TAE gel containing ethidium bromide, and their size was compared to a molecular weight marker. Amplification of β-actin, a relatively invariant internal reference RNA, was performed in parallel, and cDNA amounts were standardized to equivalent β-actin mRNA levels. These primer sets specifically recognized only the genes of interest as indicated by amplification of a single band of the expected size (500 bp for COX-2 and 514 bp for β-actin) and direct sequence analysis of the PCR products.

### Plasmid construction, transient transfection, and luciferase assays

The mouse COX-2 promoter was constructed as described previously [37] with some modifications. The upstream region (−907 to +70) of the mouse COX-2 promoter was cloned to the pGL3-basic vector containing the luciferase reporter system. Introduction of a double-point mutation into the AP-1-binding site (ACAGTCA to ACAACCA) to generate pGL-COX2-mAP1 was performed using the following (forward) primer: 5′-GGTACCGACGTACAGACCAGACACGG-3′. The underlined nucleotides indicate the positions of substituted bases. The mutant construct was cloned into the pGL3-basic vector containing the luciferase reporter system. All plasmids were prepared by using QIAGEN plasmid DNA preparation kits. The shRNA for c-Src, EGFR, p85, and Akt was provided by Dr. C.P. Tseng (Chang Gung University). The siRNAs for c-Jun and scrambled control were from Dharmacon Research Inc. (Lafayette, CO, USA), and AP-1-promoter or COX-2 promoter reporter construct was transfected into cells using the Lipofetamine-2000 transfection reagent according to the manufacturer’s instructions (Invitrogen, Carlsbad, CA). The transfection efficiency (~60%) was determined by transfection with enhanced EGFP. To assess promoter activity, cells were collected and disrupted by sonication in lysis buffer (25 mM Tris-phosphate, pH 7.8, 2 mM EDTA, 1% Triton X-100, and 10% glycerol). After centrifugation, aliquots of the supernatants were tested for luciferase activity using a luciferase assay system. Firefly luciferase activities were standardized to β-galactosidase activity.

### Chromatin immunoprecipitation (ChIP) assay

The assay was performed as described previously [38] with modifications. In brief, bEnd.3 cells were cross-linked with 1% formaldehyde for 10 min at 37 °C and washed three times with ice-cold PBS containing 1 mM phenylmethylsulfonyl fluoride (PMSF) and 1% aprotinin. Soluble chromatin was prepared using a ChIP assay kit (Upstate) according to the manufacturer’s recommendations, and immunoprecipitated without (control) or with anti-c-Jun antibody and normal goat immunoglobulin G (IgG). Following washes and elution, precipitates were heated overnight at 65 °C to reverse cross-linking of DNA and protein. DNA fragments were purified by phenol-chloroform extraction and ethanol precipitation. The purified DNA was subjected to PCR amplification using the primers specific for the region (−371 to +70) containing AP-1-binding domain present in the COX-2 promoter, sense primer: 5′-GGGGGAGGGAAGCTGTGACACTCTTGAGCTTT-3′ antisense primer: 5′-GACAGTGCTGAGATTCTTCGTGAGCAGAGTCC-3′. PCR fragments were analyzed on 2% agarose 1× TAE gel containing ethidium bromide, and the size (440 bp) was compared to a molecular weight marker.

### Measurement of PGE_2_ release

The cells were seeded in 12-well plates and grown to confluence. Cells were shifted to serum-free DMEM/F-12 medium for 24 h, and then treated with ET-1 for various time intervals. The culture supernatants were collected to measure PGE_2_ levels using an EIA kit as specified by the manufacturer (Cayman Chemical).

### Analysis of data

All data were estimated using GraphPad Prism Program (GraphPad, San Diego, CA). Quantitative data were analyzed by one-way ANOVA followed by Tukey’s honestly significant difference tests between individual groups. Data were expressed as mean ± SEM. A value of *P* < 0.05 was considered significant.

## Results

### The c-Src tyrosine kinase mediates ET-1-induced COX-2 expression in bEnd.3 cells

**Figure 1:**
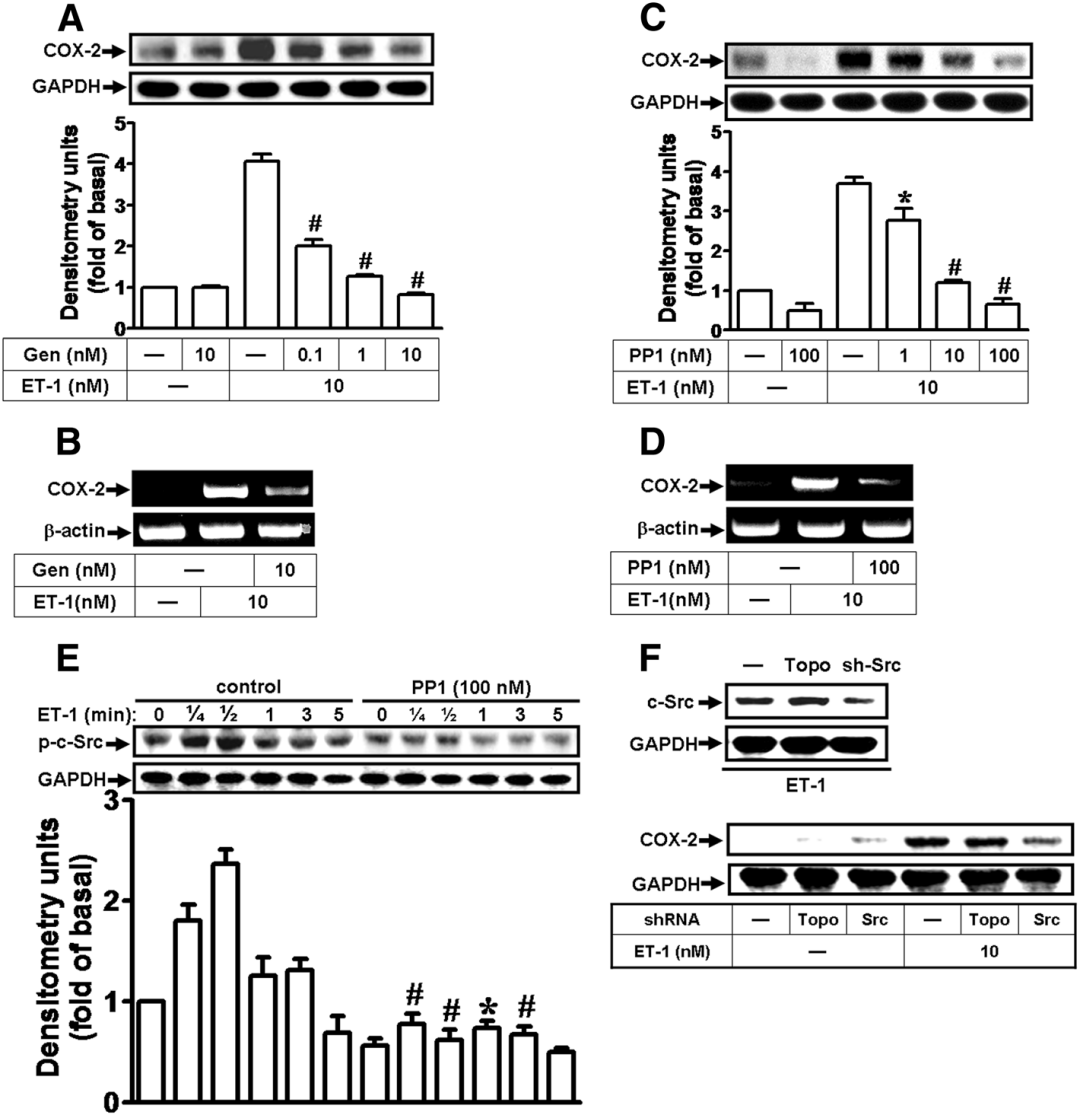
**ET-1 induces COX-2 expression via c-Src tyrosine kinase in bEnd.3 cells.** (**A**,**B**) Involvement of protein tyrosine kinases in ET-1-induced COX-2 expression; cells were pretreated with various concentrations of genistein (0.1, 1, or 10 nM) for 1 h and incubated with 10 nM ET-1 for 6 h (**A**) or 1 h (**B**). (**C**,**D**) Involvement of c-Src tyrosine kinase in ET-1-induced COX-2 expression; cells were pretreated with various concentrations of PP1 (1, 10, or 100 nM) for 1 h and incubated with 10 nM ET-1 for 6 h (**C**) or 1 h (**D**). (**E**) Time dependence of ET-1-induced c-Src phosphorylation; cells were treated with 10 nM ET-1 for the indicated time intervals in the presence or absence of PP1 (100 nM). The phosphorylation of c-Src was analyzed by Western blot using an anti-phospho-c-Src antibody. (**F**) Cells were transfected with shRNA of c-Src (sh-Src) for 24 h (Topo: as a control) and treated with 10 nM ET-1 for 6 h. The COX-2 protein (**A**,**C**,**F**) and mRNA (**B**,**D**) were analyzed by Western blotting and RT-PCR, respectively. Data are expressed as mean ± SEM of three individual experiments (*n* = 3). ^*^P < 0.05, ^#^*P* < 0.01 as compared with cells stimulated by ET-1 alone.

It has been well established that cytoplasmic tyrosine kinases of the c-Src family are involved in signaling events evoked by G protein-coupled receptors (GPCRs), which modulate many cellular functions. To determine whether c-Src is involved in ET-1-induced COX-2 expression in bEnd.3 cells, a pan-protein tyrosine kinases inhibitor genistein and a selective pharmacological inhibitor of c-Src were used. As shown in Figure 1A-D, pretreatment with genistein (Gen) or PP1 for 1 h prior to exposure to ET-1 for 6 or 1 h concentration-dependently blocked ET-1-induced COX-2 protein or mRNA expression. To further demonstrate whether ET-1 stimulates phosphorylation of c-Src, which is involved in these responses, as shown in Figure 1E, ET-1 stimulated a time-dependent phosphorylation of c-Src with a maximal response within 30 s in bEnd.3 cells. Pretreatment with PP1 (100 nM) significantly attenuated ET-1-stimulated phosphorylation of c-Src during the period of observation. To further ensure the involvement of c-Src in ET-1-induced COX-2 expression, transfection of cells with c-Src shRNA downregulated the total c-Src protein and attenuated ET-1-induced COX-2 expression (Figure 1F). The results demonstrated that ET-1-induced COX-2 expression is mediated through a c-Src-dependent pathway in bEnd.3 cells.

### ET-1 induces COX-2 expression via transactivation of EGFR

**Figure 2:**
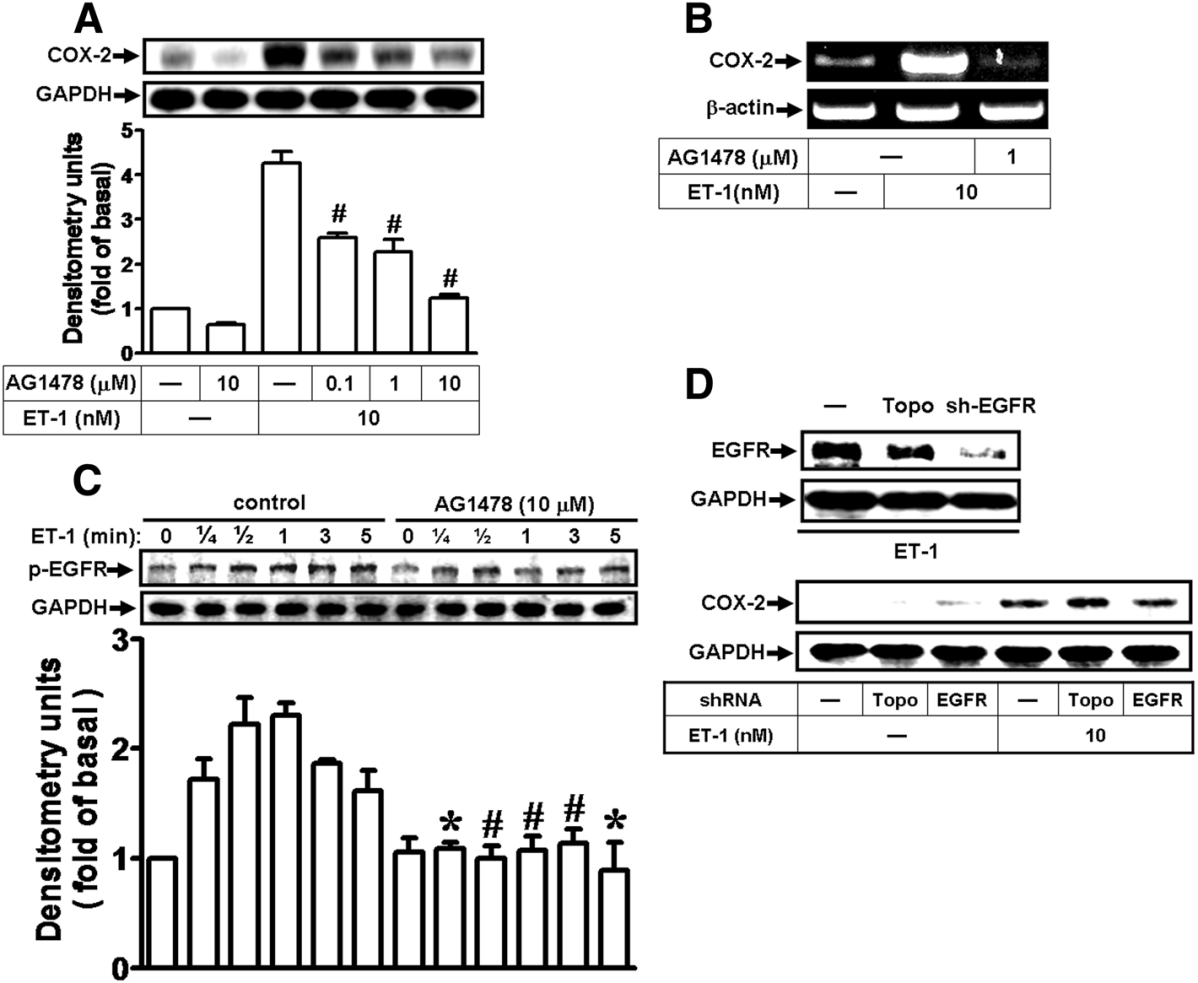
**Transactivation of EGFR is involved in ET-1-induced COX-2 expression.** (**A**,**B**) Cells were pretreated with various concentrations of AG1478 (0.1, 1, or 10 μM) for 1 h and incubated with 10 nM ET-1 for 6 h (A) or 1 h (**B**). (**C**) Time dependence of ET-1-induced EGFR phosphorylation; cells were treated with 10 nM ET-1 for the indicated time intervals in the presence or absence of AG1478 (10 μM). The phosphorylation of EGFR was analyzed by Western blot using an anti-phospho-EGFR antibody. (**D**) Cells were transfected with shRNA of EGFR (sh-EGFR) for 24 h (Topo: as a control) and incubated with 10 nM ET-1 for 6 h. The COX-2 protein (**A**,**D**) and mRNA (**B**) were analyzed by Western blotting and RT-PCR, respectively. Data are expressed as mean ± SEM of three individual experiments (*n* = 3). ^*^P < 0.05, ^#^*P* < 0.01 as compared with cells stimulated by ET-1 alone.

Cross-talk between GPCRs and RTKs has been shown to regulate the expression of several target proteins in various cell types. It has been reported that transactivation of RTKs, EGFR especially, mediates signalings activated by GPCR ligands, such as ET-1, lysophosphatidic acid, and bradykinin [27,39]. To examine whether RTK transactivation is required for ET-1-induced COX-2 expression, as shown in Figure 2A and B, pretreatment with a selective EGFR inhibitor AG1478 blocked ET-1-induced COX-2 protein and mRNA expression in a concentration-dependent manner. Furthermore, to demonstrate whether ET-1 stimulates EGFR phosphorylation, bEnd.3 cells were stimulated with ET-1 for the indicated time intervals. The data showed that ET-1 stimulated EGFR phosphorylation in a time-dependent manner with a maximal response within 30–60 s (Figure 2C). Pretreatment with AG1478 (10 μM) inhibited ET-1-stimulated EGFR phosphorylation during the period of observation. To confirm that EGFR is essential for ET-1-induced COX-2 expression, as shown in Figure 2D, transfection with EGFR shRNA downregulated the total EGFR protein and attenuated ET-1-induced COX-2 expression. The results suggested that transactivation of RTK (EGFR) is involved in ET-1-induced COX-2 expression in bEnd.3 cells.

### Involvement of PI3K/Akt cascade in ET-1-induced COX-2 expression

**Figure 3:**
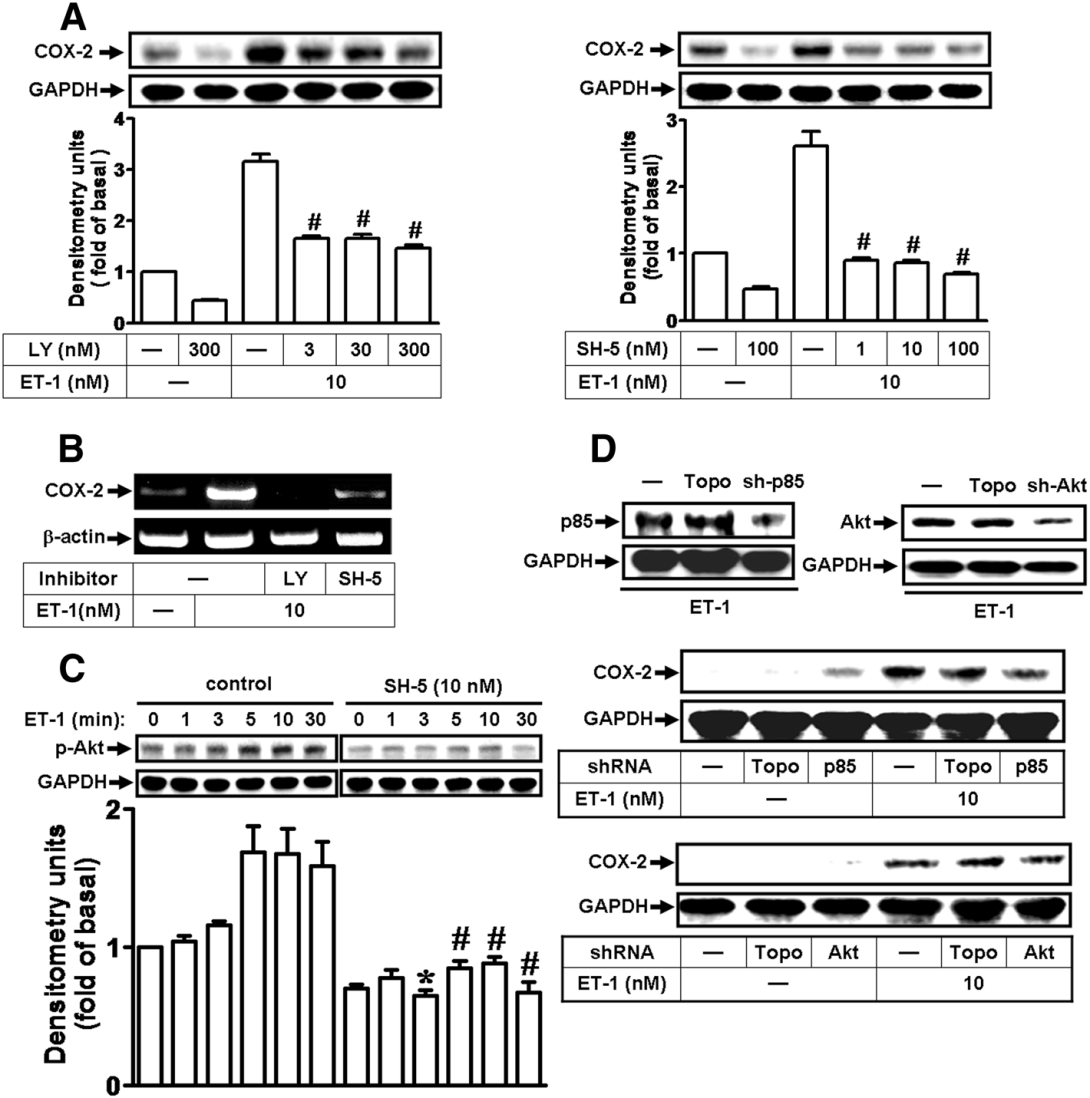
**ET-1-induced COX-2 expression is mediated through PI3K/Akt cascade.** (**A**,**B**) Cells were pretreated with various concentrations of LY294002 (3, 30, or 300 nM) or SH-5 (1, 10, or 100 nM) for 1 h, and incubated with 10 nM ET-1 for 6 h (A) or 1 h (**B**). (**C**) Time dependence of ET-1-induced Akt phosphorylation; cells were treated with 10 nM ET-1 for the indicated time intervals in the presence or absence of SH-5 (10 nM). The phosphorylation of Akt was analyzed by Western blot using an anti-phospho-Akt antibody. (**D**) Cells were transfected with p85 (sh-p85) or Akt (sh-Akt) shRNA for 24 h (Topo: as a control) and incubated with 10 nM ET-1 for 6 h. The COX-2 protein (**A**,**D**) and mRNA (**B**) were analyzed by Western blotting and RT-PCR, respectively. Data are expressed as mean ± SEM of three individual experiments (*n* = 3). ^*^P < 0.05, ^#^*P* < 0.01 as compared with cells stimulated by ET-1 alone.

Akt has been shown to be a downstream component of the EGFR pathway. Thus, we examined whether the PI3K/Akt cascade is involved in ET-1-induced responses; the inhibitors of PI3K (LY294002) and Akt (SH-5) [40] were used. As shown in Figure 3A and B, pretreatment of cells with LY294002 or SH-5 concentration-dependently attenuated COX-2 protein and mRNA induction by ET-1, implying the involvement of the PI3K/Akt cascade in ET-1-induced COX-2 expression. Next, to ensure whether ET-1 stimulates PI3K/Akt cascade activation, cells were stimulated with ET-1 for the indicated time intervals, and the activation of Akt was determined by Western blotting using an anti-phospho-Akt antibody. The data showed that ET-1 stimulated Akt phosphorylation in a time-dependent manner with a maximal response within 5–10 min (Figure 3C). Pretreatment with an Akt inhibitor SH-5 (10 nM) markedly attenuated ET-1-stimulated Akt phosphorylation during the period of observation. To further confirm the role of the PI3K/Akt cascade in ET-1-induced COX-2 expression, as shown in Figure 3D, transfection with p85 or Akt shRNA blocked the total level of p85 or Akt protein and attenuated ET-1-induced COX-2 expression. The results indicated that the PI3K/Akt cascade plays a critical role in ET-1-induced COX-2 expression in bEnd.3 cells.

### The c-Jun/AP-1 is required for ET-1-induced COX-2 expression and PGE_2_ release

**Figure 4:**
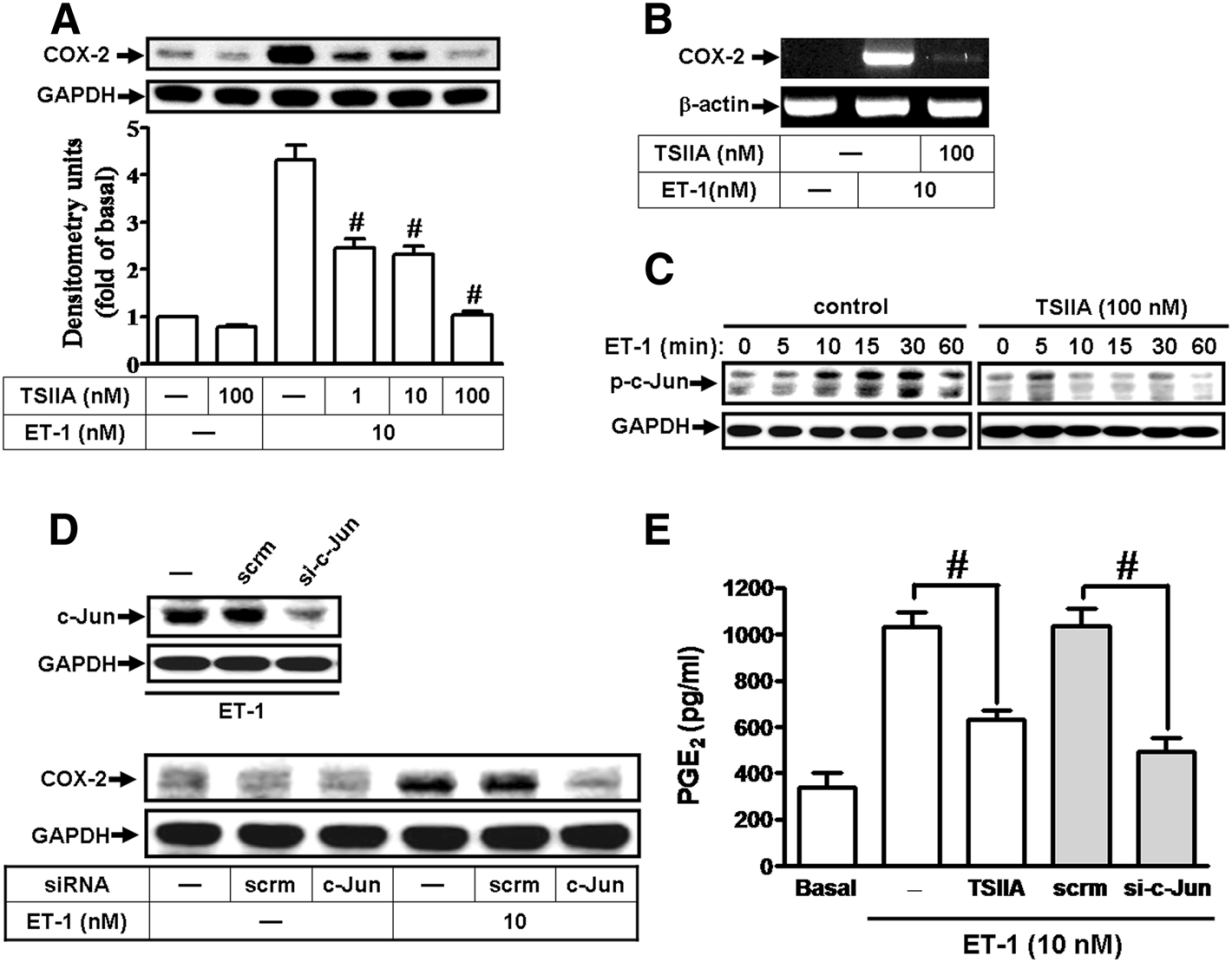
**AP-1/c-Jun is essential for ET-1-induced COX-2 expression.** (**A**,**B**) Cells were pretreated with various concentrations of tanshinone IIA (TSIIA, 1, 10, or 100 nM) for 1 h and incubated with 10 nM ET-1 for 6 h (**A**) or 1 h (**B**). (**C**) Time dependence of ET-1-induced c-Jun/AP-1 phosphorylation; cells were treated with 10 nM ET-1 for the indicated time intervals in the presence or absence of TSIIA (100 nM). The phosphorylation of c-Jun/AP-1 was analyzed by Western blot using an anti-phospho-c-Jun antibody. (**D**) Cells were transfected with siRNA of c-Jun (si-c-Jun) or scrambled (scrm; as a control) for 24 h and incubated with 10 nM ET-1 for 6 h. The COX-2 protein (**A**,**D**) and mRNA (**B**) were analyzed by Western blotting and RT-PCR, respectively. (**E**) Involvement of c-Jun/AP-1 in PGE_2_ release induced by ET-1; cells were pretreated with TSIIA (100 nM) or transfected with c-Jun siRNA (si-c-Jun) and then incubated with ET-1 (10 nM) for 6 h. The conditioned media were collected to assay PGE_2_ level by EIA kit. Data are expressed as mean ± SEM of three individual experiments (*n* = 3). ^#^*P* < 0.01 as compared with cells stimulated by ET-1 alone.

It is well known that the COX-2 promoter consists of AP-1 binding sites [33]. Therefore, we determined whether the transcription factor AP-1 is involved in ET-1-induced COX-2 expression in bEnd.3 cells; an AP-1 inhibitor tanshinone IIA (TSIIA) was used. The data showed that pretreatment with TSIIA significantly inhibited ET-1-induced COX-2 protein and mRNA expression in a concentration-dependent manner (Figure 4A and B). AP-1 consists of homodimers of the Jun family or heterodimers of Jun and Fos family proteins [41]. Here we further investigated whether ET-1 stimulates AP-1 activation through regulating phosphorylation of c-Jun/AP-1; as shown in Figure 4C, ET-1 stimulated a time-dependent phosphorylation of c-Jun with a maximal response within 15–30 min in bEnd.3 cells, which was attenuated by pretreatment with TSIIA during the period of observation. Subsequently, to further demonstrate that c-Jun is involved in the response, indeed, a siRNA of c-Jun was used. The data showed that transfection of cells with c-Jun siRNA downregulated the c-Jun protein expression and markedly attenuated ET-1-induced COX-2 expression (Figure 4D). We further determined whether c-Jun/AP-1 is also involved in ET-1-induced PGE_2_ release; as shown in Figure 4E, pretreatment with TSIIA or transfection with c-Jun siRNA significantly attenuated PGE_2_ release induced by ET-1, suggesting that c-Jun/AP-1 participates in ET-1-induced COX-2 expression and PGE_2_ release in bEnd.3 cells.

### ET-1 stimulates c-Src-dependent transactivation of EGFR/PI3K/Akt leading to MAPKs and c-Jun phosphorylation

**Figure 5:**
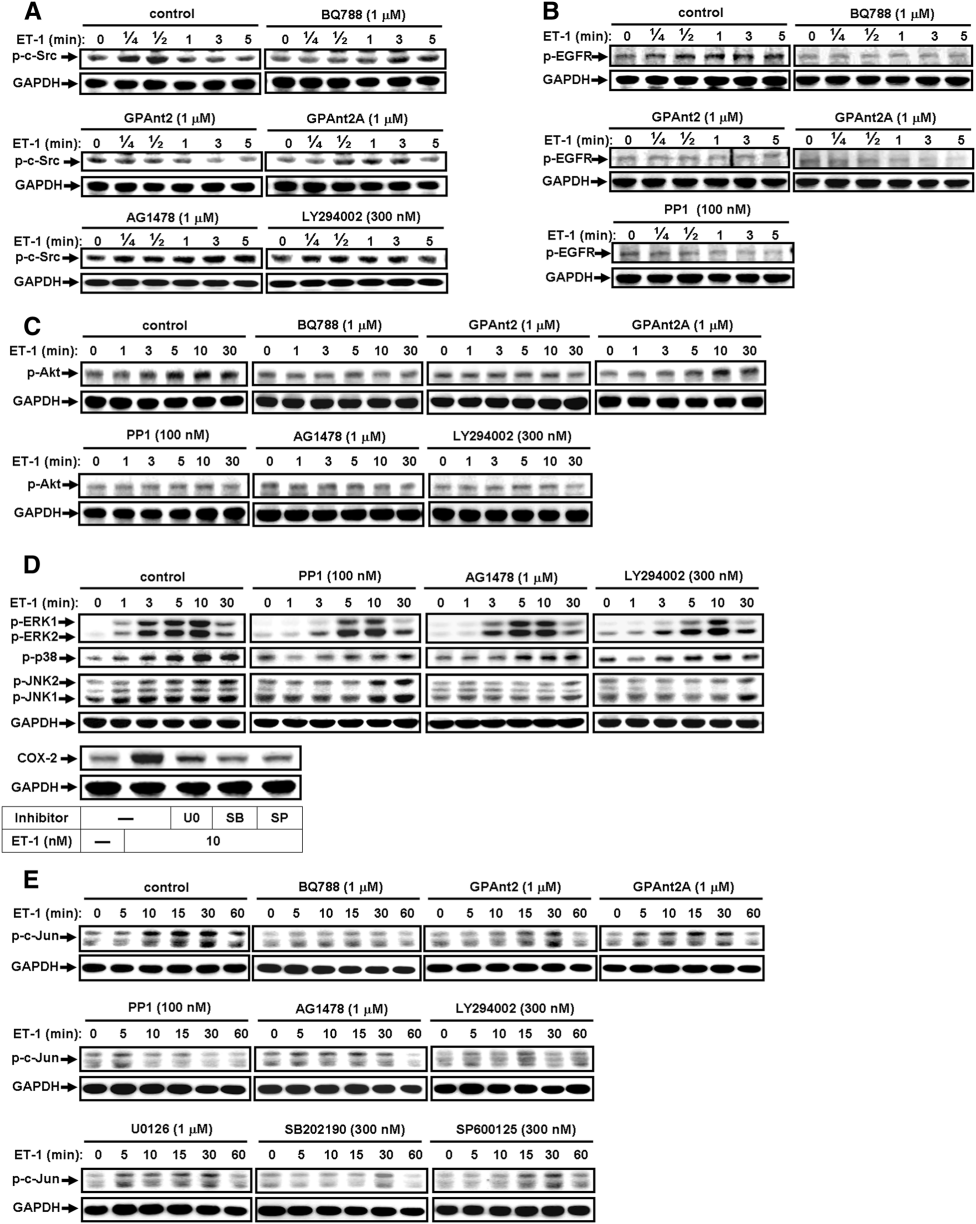
**ET-1 stimulates c-Src-dependent transactivation of EGFR/PI3K/Akt leading to MAPKs and c-Jun phosphorylation.** (**A**) For c-Src phosphorylation, cells were incubated with 10 nM ET-1 for the indicated time intervals in the absence or presence of BQ788 (1 μM), GPAnt2 (1 μM), GPAnt2A (1 μM), AG1478 (1 μM), or LY294002 (300 nM). (**B**) For EGFR phosphorylation, cells were incubated with 10 nM ET-1 for the indicated time intervals in the absence or presence of BQ788, GPAnt2, GPAnt2A, or PP1 (100 nM). (**C**) For Akt phosphorylation, cells were incubated with 10 nM ET-1 for the indicated time intervals in the absence or presence of BQ788, GPAnt2, GPAnt2A, PP1, AG1478, or LY294002. (**D**) For MAPKs phosphorylation, cells were incubated with 10 nM ET-1 for the indicated time intervals in the absence or presence of PP1, AG1478, or LY294002. For COX-2 expression, cells were incubated with ET-1 (10 nM) for 6 h in the absence or presence of U0126 (1 μM), SB202190 (300 nM), or SP600125 (300 nM). (**E**) For c-Jun phosphorylation, cells were incubated with 10 nM ET-1 for the indicated time intervals in the absence or presence of BQ788, GPAnt2, GPAnt2A, PP1, AG1478, LY294002, U0126 (1 μM), SB202190 (300 nM), or SP600125 (300 nM). The cell lysates were collected and analyzed by Western blotting using an anti-phospho-c-Src, anti-phospho-EGFR, anti-phospho-Akt, anti-phospho-ERK1/2, anti-phospho-p38 MAPK, anti-phospho-JNK1/2, anti-phospho-c-Jun, or anti-GAPDH (as an internal control) antibody. The figure represents one of three similar experiments (*n* = 3).

We have demonstrated that ET-1-induced COX-2 expression is mediated through activation of c-Src, EGFR, PI3K/Akt, or c-Jun/AP-1. Thus, we made an attempt to sequentially differentiate the signaling pathway of these molecules. First, cells were pretreated with antagonists of ET_B_ receptor (BQ788) or G proteins (e.g., GPAnt2 for G_i_ or G_s_ protein and GPAnt2A for G_q_ protein) [42] for 1 h prior exposure to ET-1 for the indicated time intervals. As shown in Figure 5A, ET-1-stimulated c-Src phosphorylation was significantly attenuated by pretreatment with BQ788 (1 μM), GPAnt2 (1 μM), and GPAnt2A (1 μM), but not AG1478 and LY294002, suggesting that phosphorylation of c-Src by ET-1 was mediated through a G_i_ and G_q_ protein-coupled ET_B_ receptor. Next, pretreatment with BQ788, GPAnt2, GPAnt2A, or PP1 (100 nM) all markedly inhibited ET-1-stimulated EGFR phosphorylation at tyrosine 845 (Tyr^845^) residue, which was one of the phosphorylation sites by c-Src kinases during the observation period (Figure 5B). These results suggested that ET-1-transactivated EGFR may be mediated through a G_i_ and G_q_ protein-coupled ET_B_ receptor linking to c-Src-dependent cascade. Furthermore, we demonstrated whether the EGFR downstream signaling molecule Akt could be activated by the same pathway; cells were pretreated with BQ788, GPAnt2, GPAnt2A, PP1, AG1478 (1 μM), or LY294002 (300 nM) for 1 h and then stimulated with ET-1 for the indicated time intervals. As shown in Figure 5C, ET-1-stimulated Akt phosphorylation was attenuated by pretreatment with these pharmacological inhibitors, suggesting that phosphorylation of Akt by ET-1 was mediated via c-Src-dependent transactivation of EGFR. Furthermore, our previous reports have demonstrated that thrombin or BK stimulates activation of MAPKs via a c-Src-dependent EGFR transactivation in vascular smooth muscle cells [4,31]. Thus, to further determine whether ET-1 stimulates activation of MAPKs via c-Src-dependent EGFR transactivation, cells were pretreated with PP1, AG1478, or LY294002 before exposure to ET-1 for the indicated time intervals. As expected, we found that ET-1 stimulated phosphorylation of MAPKs, including ERK1/2, p38 MAPK, and JNK1/2, in a time-dependent manner (Figure 5D). Moreover, pretreatment with PP1, AG1478, or LY294002 significantly attenuated ET-1-stimulated phosphorylation of ERK1/2, p38 MAPK, and JNK1/2 (Figure 5D), suggesting that ET-1 stimulates MAPKs phosphorylation via a c-Src-dependent EGFR/PI3K/Akt cascade. Pretreatment with the inhibitor of ERK (U0126, 1 μM), p38 MAPK (SB202190, 300 nM), or JNK (SP600125, 300 nM) markedly attenuated ET-1-induced COX-2 expression (Figure 5D, lower panel), suggesting that ET-1-induced COX-2 expression is mediated through these MAPKs. We further demonstrated whether activation of c-Jun/AP-1 by ET-1 was also mediated through c-Src-dependent transactivation of EGFR/PI3K/Akt and MAPKs in b.End.3 cells. As shown in Figure 5E, pretreatment with these inhibitors as described above markedly attenuated ET-1-stimulated phosphorylation of c-Jun during the period of observation, suggesting that ET-1 stimulated c-Jun phosphorylation via the same pathway. These results indicated that ET-1-induced MAPKs-dependent c-Jun phosphorylation was mediated through c-Src-dependent transactivation of EGFR/PI3K/Akt in bEnd.3 cells.

### ET-1 stimulates AP-1 activation and recruitment of AP-1 to COX-2 gene promoter via c-Src-dependent EGFR/PI3K/Akt/MAPKs pathway

**Figure 6:**
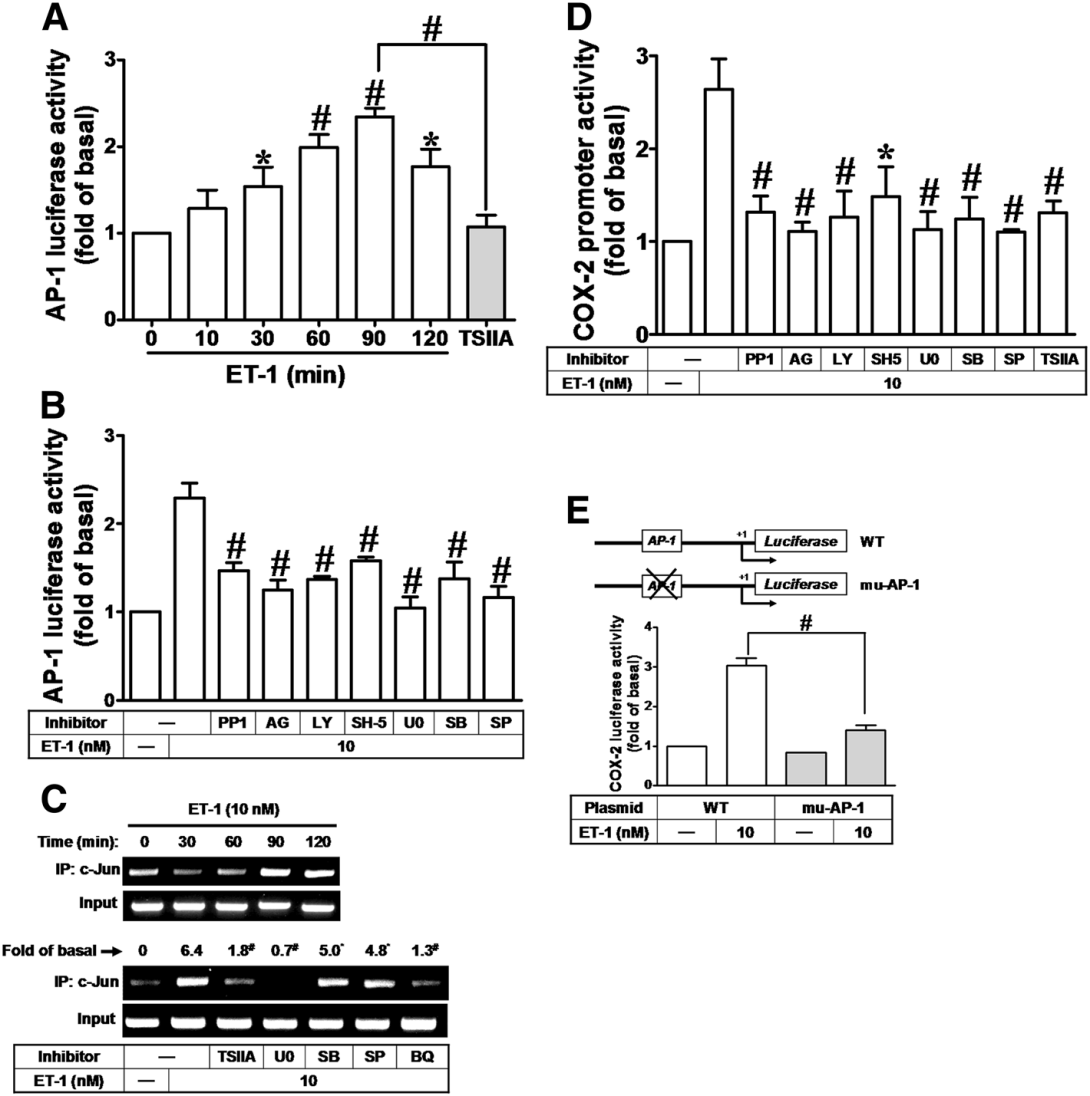
**ET-1-stimulated COX-2 promoter activity is mediated through AP-1-dependent pathway.** (**A**) Time dependence of ET-1-enhanced AP-1 transcription activity; cells were transfected with an AP-1-luciferase reporter gene and then exposed to ET-1 for the indicated time intervals. (**B**) After transfection with AP-1-luciferase reporter gene, the cells were pretreated with PP1 PP1 (100 nM), AG1478 (AG, 1 μM), LY294002 (LY, 300 nM), SH-5 (10 nM), U0126 (U0, 1 μM), SB202190 (SB, 300 nM), SP600125 (SP, 300 nM), or (**A**) TSIIA (100 nM) for 1 h and then incubated with ET-1 (10 nM) for 90 min. (**C**) Cells were pretreated without or with TSIIA, U0126 (U0), SB202190 (SB), SP600125 (SP), or BQ788 (BQ) for 1 h and then incubated with ET-1 (10 nM) for the indicated time intervals (upper panel) or 90 min (lower panel). The c-Jun/AP-1 binding activity was analyzed by chromatin-IP (ChIP)-PCR assay. (**D**) For COX-2 promoter activity, cells were pretreated with PP1, AG1478 (AG), LY294002 (LY), SH-5, U0126 (U0), SB202190 (SB), or SP600125 (SP), or TSIIA for 1 h and then incubated with ET-1 (10 nM) for 6 h. (**E**) Schematic representation of a 5′-promoter regions of the mouse different COX-2 promoter constructs, either wild-type (WT) or mutated by single-point mutation of the AP-1 binding site (mu-AP-1) cloned to the pGL-luciferase reporter gene; the translational start site (+1) of the luciferase reporter gene is indicated by an arrow. Cells were transfected with WT COX-2 promoter reporter gene (WT) or AP-1 mutated COX-2 promoter reporter gene (mu-AP-1) and then incubated with or without ET-1 (10 nM) for 6 h. The promoter reporter activity was determined. Data are expressed as mean ± SEM of at least three individual experiments (*n* = 3). ^*^P < 0.05, ^#^*P* < 0.01 as compared with ET-1 alone.

To investigate whether ET-1-stimulated AP-1 transcription activity is also mediated through this c-Src-dependent pathway, the AP-1 promoter reporter construct was used. As shown in Figure 6A, ET-1 (10 nM) stimulated an AP-1-luciferase activity increase in a time-dependent manner with a maximal response within 90 min, which was attenuated by pretreatment with TSIIA (100 nM) [43]. Furthermore, pretreatment with PP1, AG1478, LY294002, SH-5, U0126, SB202190, or SP600125 also attenuated ET-1-stimulated AP-1 transcriptional activity (Figure 6B). It has been shown that the COX-2 promoter region contains AP-1 binding sites. Hence, we used a ChIP-PCR assay to determine whether ET-1-stimulated recruitment of c-Jun/AP-1 to the COX-2 promoter is involved in COX-2 gene expression. We designed a pair of primers for the COX-2 promoter (−371 to +70) region, containing an AP-1 binding site. Chromatin was immunoprecipitated using an anti-c-Jun antibody, and the COX-2 promoter region was amplified by PCR. As shown in Figure 6C (upper part), ET-1 stimulated *in vivo* binding of c-Jun to the COX-2 promoter in a time-dependent manner with a maximal response within 90 min, which was attenuated by pretreatment with TSIIA, U0126, SB202190, SP600125, or BQ788 (Figure 6C, lower part).

We next examined whether ET-1-induced COX-2 promoter activity is also regulated by these signaling pathways. ET-1-stimulated increase in COX-2 promoter activity was attenuated by pretreatment with PP1, AG1478, LY294002, SH-5, U0126, SB202190, SP600125, or TSIIA (Figure 6D), suggesting that ET-1-induced COX-2 promoter activity is mediated through c-Src-dependent EGFR/PI3K/Akt/MAPKs and c-Jun/AP-1 in bEnd.3 cells. To further ensure that AP-1 is involved in ET-1-induced COX-2 promoter activity via binding to the AP-1 binding element on the COX-2 promoter region, the wild-type COX-2 promoter mutated by single-point mutation of the AP-1 binding site (mu-AP-1) was constructed (as illustrated in Figure 6E, upper part). ET-1-stimulated COX-2 promoter activity was significantly blocked in cells transfected with an mt-AP1-COX-2 reporter construct (Figure 6E, lower part). These results confirmed that ET-1-induced COX-2 promoter activity is mediated through binding of AP-1 (c-Jun) to the AP-1 element of the COX-2 promoter region.

### ET-1-induced PGE_2_ release is mediated through c-Src-dependent transactivation of EGFR

**Figure 7:**
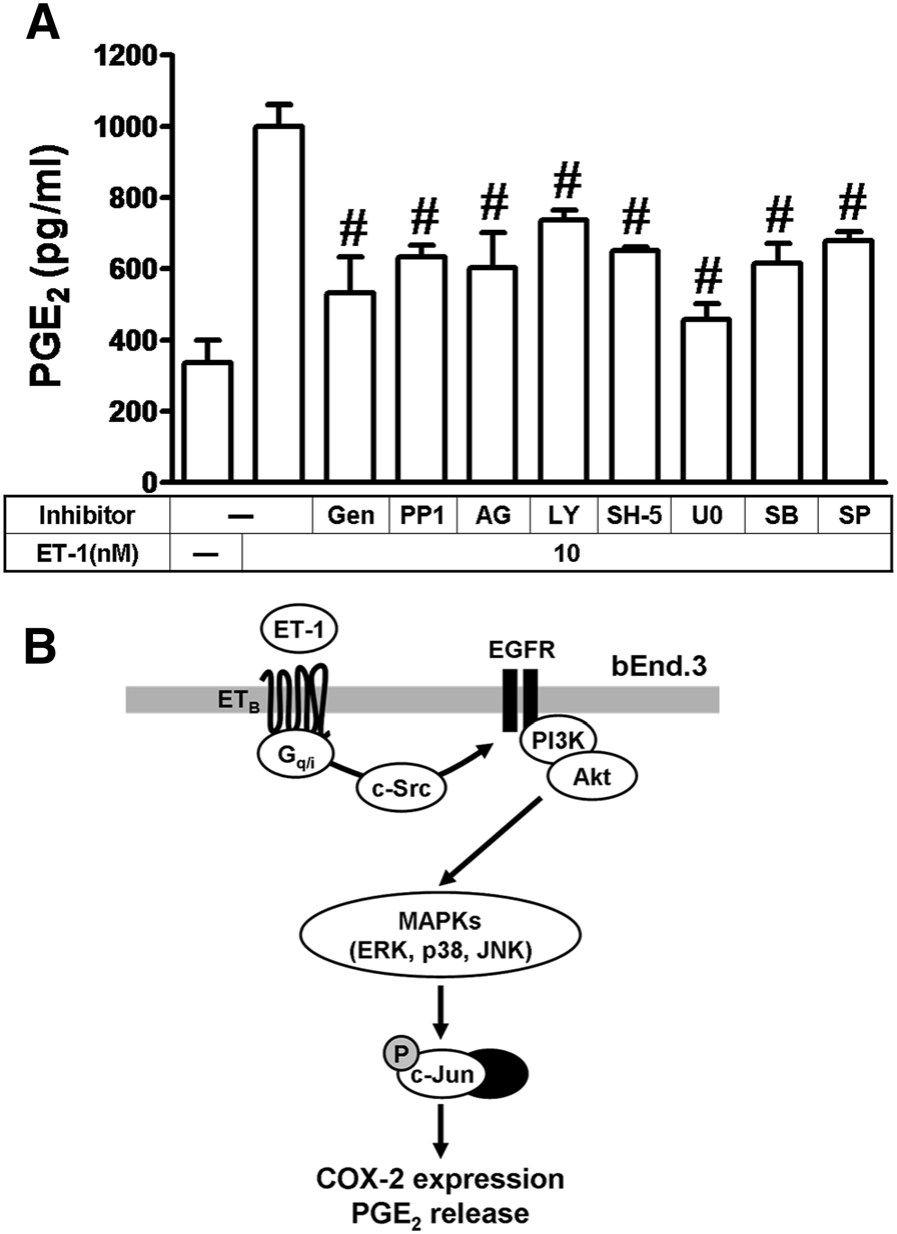
**ET-1-induced PGE**_**2**_**release is mediated through a c-Src-dependent transactivation of EGFR/PI3K/Akt cascade.** (**A**) Cells were incubated with 10 nM ET-1 for 6 h in the absence or presence of genistein (Gen, 10 nM), PP1 (100 nM), AG1478 (AG, 1 μM), LY294002 (LY, 00 nM), SH-5 (100 nM), U0126 (U0, 1 μM), SB202190 (SB, 300 nM), or SP600125 (SP, 300 nM), and the conditioned media were collected to assay the PGE_2_ level by EIA kit. Data are expressed as mean ± SEM of three individual experiments (*n* = 3). ^#^*P* < 0.01, as compared with cells stimulated by ET-1 alone. (**B**) Schematic representation of signaling pathways involved in ET-1-induced COX-2 expression in bEnd.3 cells. Binding of ET-1 to G_i_ and G_q_ protein-coupled ET_B_ receptors results in c-Src-dependent transactivation of EGFR/PI3K/Akt linking to MAPKs (e.g., ERK, p38 MAPK, and JNK) and c-Jun/AP-1. The COX-2 transcription is dependently regulated by MAPK-dependent AP-1 pathways. These signaling pathways contribute to sustained activation of AP-1 required for COX-2 expression and PGE_2_ release in bEnd.3 cells.

Ultimately, to demonstrate the functional activity of upregulated COX-2 by ET-1 on bEnd.3 cells, we evaluated the PGE_2_ release by EIA kit assay. The levels of PGE_2_ release were measured at 6 h induced by ET-1 (10 nM). ET-1-induced PGE_2_ release was significantly blocked by pretreatment with genistein (Gen, 1 μM), PP1 (10 nM), AG1478 (AG, 10 μM), LY294002 (LY, 300 nM), SH-5 (10 μM), U0126 (U0, 1 μM), SB202190 (SB, 300 nM), or SP600125 (SP, 300 nM) (Figure 7A), suggesting that upregulation of COX-2 is mediated through c-Src-dependent transactivation of EGFR/PI3K/Akt/MAPKs pathway associated with PGE_2_ released by ET-1 in bEnd.3 cells.

## Discussion

ET-1 is elevated in the regions of vascular injuries and inflammation [9,19,44]. Circumstantial evidence has further demonstrated that overexpression of ET-1 on endothelial cells has deleterious effects on ischemic brain [7,10,11]. Additionally, ET-1 has been shown to upregulate the expression of COX-2 through MAPKs in various cell types [25,26,45]. The upregulation of COX-2 has been shown in several inflammatory diseases and displays a wide range of biological activities in different tissues, blood vessels in particular, including development, proliferation, cancers, and inflammation [1,2]. Several studies have also demonstrated that high levels of PGs, synthesized by inducible COX-2, are involved in inflammatory responses. However, the mechanisms of ET-1-induced COX-2 expression in brain endothelial cells remain unclear. Herein we used cultured models of mouse brain endothelial cell line (bEnd.3) and applied Western blot analysis, selective pharmacological inhibitors, transfection with shRNA or siRNAs, ChIP-PCR, and promoter reporter assay to investigate the signaling pathways underlying ET-1-induced COX-2 expression and PGE_2_ release. Our results demonstrated that in bEnd.3 cells activation of ET_B_ receptor-mediated c-Src-dependent transactivation of EGFR, PI3K/Akt, MAPKs (ERK, p38 MAPK, and JNK), and the AP-1 signaling cascade is essential for ET-1-induced COX-2 gene expression and PGE_2_ release.

Several studies have found that an agonist of GPCR coupling to different G proteins transactivates RTKs such as EGFR in diverse cell types and sequential linking to activation of downstream signals such as MAPKs [19,21,22]. We have demonstrated a significant expression of ET_B_ receptor in bEnd.3 cells by RT-PCR. Hence, in this study, the involvement of ET_B_ receptors in these responses was confirmed because pretreatment with BQ-788 (an ET_B_ receptor antagonist) reduced the ET-1-stimulated phosphorylation of c-Src, EGFR, Akt, MAPKs (i.e., ERK, p38 MAPK, JNK), and c-Jun/AP-1 (Figure 5), but not by an ET_A_ receptor antagonist BQ-123 (data not shown), suggesting that the ET_B_ receptor predominantly mediates ET-1 stimulation in these responses in bEnd.3 cells. Next, several subtypes of G proteins are potentially implicated in ET-1-induced COX-2 expression. We used GPA2 (a G_i/o_ protein antagonist) and GPA2A (a G_q_ protein antagonist) to interrupt G protein signaling and the consequent phosphorylation of these signaling molecules (Figure 5), indicating that ET-1-stimulated c-Src-dependent transactivation of EGFR is mediated by a GPCR (i.e., ET_B_) coupling to either G_i_ or G_q_ protein in bEnd.3 cells, consistent with previous studies from esophageal smooth muscle cells [45] and rat brain astrocytes [20]. In contrast, a report shows that ET-1 induced COX-2 expression via ET_A_ receptors in peripheral lung microvascular smooth muscle cells [25]. However, in respiratory and cardiovascular systems, both ET receptor subtypes, ET_A_ especially, are involved in progression of airway [46] and cardiovascular diseases [44]. These differences may be due to cell-type-specific or different experimental conditions.

It has been reported that transactivation of RTK, EGFR especially, occurs in response to activation of many GPCRs such as endothelin-1 [27,32,39]. Several lines of evidence have also shown that the βγ complex of G_i_ protein activates non-RTKs, such as the c-Src family, which might transactivate RTKs and modulate various cellular functions [47,48]. Moreover, the involvement of c-Src in the transactivation of EGFR by GPCRs has been reported in various cell types [47,49]. Although transactivation of EGFR by GPCR agonists has been well studied, the signaling mechanism by which ET-1-stimulated transactivation of RTK such as EGFR in brain microvascular endothelial cells has not been completely understood. Thus, in this study, we investigated whether protein tyrosine kinase pathways, such as c-Src-dependent transactivation of EGFR, are involved in ET-1-induced COX-2 expression. First, our data demonstrated that ET-1-induced COX-2 expression is mediated through protein tyrosine kinases including c-Src and EGFR by pharmacological inhibitors and transfection with a c-Src or EGFR shRNA, which all significantly inhibited induction of COX-2 gene expression by ET-1 (Figures 1 and 2). Next, we found that ET-1 can stimulate phosphorylation of c-Src and EGFR via G_i_ and G_q_ protein-coupled ET_B_ receptors (Figure 5A and B), and following the PP1 significantly inhibited c-Src and EGFR phosphorylation (Figures 1E and 5B) and subsequent upregulation of COX-2 (Figure 1C and D) and PGE_2_ release (Figure 7A). These results demonstrated that c-Src-dependent EGFR transactivation plays a critical role in ET-1-induced COX-2 expression and PGE_2_ release, consistent with previous reports showing the involvement of EGFR transactivation in ET-1-induced cell proliferation in ovarian cancer cells [29] and thrombin- or ET-1-induced COX-2 expression in VSMCs [4,30,44].

Abnormal MAPK regulation might be implicated in several models of CNS injury and inflammation [50]. Several lines of evidence demonstrate that MAPKs could be activated by GPCR agonists by different signal pathways [23]. MAPKs activation by ET-1 has been shown to modulate various cellular responses in various cell types [20,24]. Thus, activation of MAPKs may be implicated in the expression of inflammatory genes in several models of vascular injury and inflammation [4,51]. Additionally, an agonist of GPCR has been found to transactivate EGFRs in diverse cell types and shows sequential linking to MAPK activation, ERK1/2 especially [19,21,22]. Our data showed that ET-1 stimulated c-Src-dependent transactivation of the EGFR and PI3K/Akt cascade (Figure 5A-C), and pretreatment with PP1, AG1478, or LY294002 attenuated phosphorylation of MAPKs, including ERK1/2, p38 MAPK, and JNK1/2 (Figure 5D), suggesting that ET-1-stimulated activation of MAPKs is mediated through c-Src-dependent transactivation of the EGFR and PI3K/Akt cascade. Moreover, ET-1-induced COX-2 expression was mediated through MAPKs (Figure 5D, lower panel). Although most studies indicate that activation of EGFR may lead to ERK1/2 activation via a Grb2/Sos/Ras/Raf cascade, increasing evidence demonstrates that transactivation of the EGFR/PI3K/Akt cascade also plays a critical role in the activation of ERK1/2. Our results are consistent with previous reports indicating that BK mediates cell proliferation or thrombin induces COX-2 expression via transactivation of the EGFR and ERK1/2 cascade in VSMCs [4,31], and thrombin stimulates cell migration in SMCs [52]. In contrast, many studies suggest that thrombin-induced mitogenic action in astrocytes or VSMCs occurs independently of EGFR transactivation [53,54]. Regarding the MAPKs, our results are the first to show that p38 MAPK and JNK1/2 play a critical role in the induction of COX-2 by ET-1 in brain microvascular endothelial cells.

It has been well established that inflammatory responses following exposure to extracellular stimuli are highly dependent on activation of AP-1 transcription factor, which plays an important role in the regulation of several gene expressions [41]. The 5′-flanking region of the COX-2 promoter has been shown to contain several binding sequences for various transcription factors including AP-1 [33,35]. Therefore, the regulation of COX-2 transcription may be mediated by aberrant activation of several distinct transcription factors dependent on agonists [26,55]. These studies suggest that AP-1 plays a critical role in the regulation of COX-2 expression in the development of inflammatory responses. Our data showed that ET-1-induced COX-2 gene expression and PGE_2_ release were significantly abolished by an AP-1 inhibitor tanshinone IIA (TSIIA) [43] (Figure 4A, B, and E) or c-Jun (a AP-1 subunit) siRNA (Figure 4D and E), suggesting that c-Jun/AP-1 is involved in ET-1-induced COX-2 expression in bEnd.3 cells. Moreover, ET-1-stimulated c-Jun phosphorylation (Figure 4C) and AP-1-Luc transcriptional activity (Figure 6A) were significantly inhibited by TSIIA and three MAPK inhibitors U0126 (MEK1/2), SB202190 (p38 MAPK), or SP600125 (JNK1/2) (Figures 4C5E6A, and 6B). Here, we found the inhibitory effect of TSIIA on ET-1-stimulated c-Jun phosphorylation in bEnd.3 cells, which is consistent with our recent study in brain astrocytes [56]. Our data further showed that these ET-1-stimulated responses were significantly blocked by PP1, AG1478, and LY294002 in these cells (Figures 5E6B, and 6D). These findings suggested that ET-1-induced COX-2 expression and PGE_2_ release are mediated through an AP-1-dependent mechanism via c-Src-dependent transactivation of EGFR, PI3K/Akt, and MAPK cascades. These findings are consistent with recent studies indicating that COX-2 expression induced by phorbol ester (TPA) was mediated by JNK1/2 and AP-1 activation in human breast epithelial cell line (MCF-10A) [57] and COX-2 expression induced by EV-71 via p42/p44 MAPK linking to AP-1 activation in rat brain astrocytes [58]. The involvement of AP-1 in ET-1-induced COX-2 expression is also consistent with previous a report indicating that ET-1-stimulated activation of AP-1 regulates expression of other target genes involved in various CNS inflammatory processes [20].

## Conclusions

In this study, we demonstrated that the ET-1/ET receptor system exerts its inducing effects on COX-2 gene expression and PGE_2_ release in mouse cultured brain endothelial (bEnd.3) cells. The G_i_ and G_q_ protein-coupled ET_B_ receptor, c-Src-dependent transactivation of EGFR, PI3K/Akt, ERK1/2, p38 MAPK, JNK1/2, and c-Jun/AP-1 cascades cooperatively mediated these effects of ET-1. Based on the observations from the literature and our findings, Figure 7B depicts a model for the signaling mechanisms implicated in ET-1-induced COX-2 gene expression in mouse-cultured bEnd.3 cells. These findings concerning ET-1-induced COX-2 expression and PGE_2_ generation imply that ET-1 might play a critical role in brain injury, vascular inflammation, and CNS disorders, mediated by c-Src-dependent transactivation of EGFR linking to MAPKs/AP-1 pathways in brain microvascular endothelial cells. Pharmacological approaches suggest that targeting COX-2 and its upstream signaling components may provide useful therapeutic strategies for brain injury and inflammatory diseases.

## Competing interests

The authors declare that they have no competing interests.

## Authors’ contributions

HLH and CCL designed and performed the experiments, acquisition and analysis of data, and drafted the manuscript. HJC and Caleb MY helped to perform experiments and prepare the manuscript. CMY conceived of the study, participated in its design and coordination, been involved in drafting the manuscript and revising it critically for important intellectual content, and gave final approval of the version to be published. All authors have read and approved the final version of this manuscript.
